# Anti-Inflammatory Activity of Chrysophanol through the Suppression of NF-κB/Caspase-1 Activation *in Vitro* and *in Vivo*

**DOI:** 10.3390/molecules15096436

**Published:** 2010-09-16

**Authors:** Su-Jin Kim, Min-Cheol Kim, Byong-Joo Lee, Dae-Hee Park, Seung-Heon Hong, Jae-Young Um

**Affiliations:** 1 Departmentof Pharmacology, College of Oriental Medicine, Institute of Oriental Medicine, Kyung Hee University, 1 Hoegi-Dong, Dongdaemun-Gu, Seoul, 130-701, Korea; E-Mails: ksj1009@khu.ac.kr (S-J.K.); nlbo92@hanmail.net (B.-J.L.); neorufa@nate.com (D-H.P.); 2 Department of Oriental Pharmacy, College of Pharmacy, VestibuloCochlear Research Center of Wonkwang University, Iksan, Jeonbuk, Korea; E-Mails: mchol@naver.com (M.-C.K.); jooklim@wku.ac.kr (S.-H.H.)

**Keywords:** chrysophanol, inflammation, colitis, macrophage

## Abstract

Chrysophanol is a member of the anthraquinone family and has multiple pharmacological effects, but the exact mechanism of the anti-inflammatory effects of chrysophanol has yet to be thoroughly elucidated. In this study, we attempted to determine the effects of chrysophanol on dextran sulfate sodium (DSS)-induced colitis and lipopolysaccharide (LPS)-induced inflammatory responses in mouse peritoneal macrophages. The findings of this study demonstrated that chrysophanol effectively attenuated overall clinical scores as well as various pathological markers of colitis. Additionally, chrysophanol inhibited the production of tumor necrosis factor (TNF)-α, interleukin (IL)-6 and the expression of cyclooxygenase (COX)-2 levels induced by LPS. We showed that this anti-inflammatory effect of chrysophanol is through suppression of the activation of NF-κB and caspase-1 in LPS-stimulated macrophages. These results provide novel insights into the pharmacological actions of chrysophanol as a potential molecule for use in the treatment of inflammatory diseases.

## 1. Introduction

Ulcerative colitis (UC) is a typical inflammatory intestinal disease belonging to the inflammatory bowel diseases (IBD). Unfortunately, despite many years of extensive research implicating immune dysfunction, genetic susceptibility, and bacterial flora within the intestinal environment as possible factors associated with development of the disease, its pathogenesis is still poorly understood. 

Inflammation is a process that involves the action of multiple factors within a complex network. The ingress of leukocytes into the inflammation site is important in the pathogenesis of inflammatory conditions. Macrophage activation has been determined to perform an important role in the inflammatory process [1,2] and to generate potent pro-inflammatory cytokines such as tumor necrosis factor (TNF)-α, and interleukin (IL)-6, which induce inflammation and recruit other immune cells, including neutrophils [1]. Although TNF-α and IL-6 are beneficial to host defenses, they can also trigger pathological conditions when expressed in excess quantities [3]. For example, massive stimulation of macrophages after a severe Gram-negative bacterial infection can result in excessive production of pro-inflammatory cytokines and the development of fatal septic shock syndrome, as well as multiple organ failure [4]. It was reported that mucosa from patients with UC indeed have shown increased expression of IL-1 and IL-6 and proposed to play an integral role in its pathogenesis [5,6,7].Hence, there is currently a strong interest in agents that can block the generation or activities of inflammatory cytokines.

Cyclooxygenases (COX) have been implicated in a number of physiological events, including the progression of inflammation, immunomodulation, and transmission of pain. Two COX isoenzymes were identified. COX-1, the constitutive enzyme, makes prostaglandins which protects the stomach and kidney from damage. Whereas COX-2 normally is expressed at very low levels, it is rapidly induced by a variety of stimuli, such as cytokines, growth factors, hormones and carcinogens, and is believed to be responsible for the production of prostaglandins associated with mediation of inflammation. It is reported that COX-2 expression is increased in inflamed mucosa of patients with UC [8,9].

Nuclear factor-kappa B (NF-κB) performs a crucial function in the expression of many genes involved in immune and inflammatory responses. Inactive NF-κB complexes are sequestered in the cytoplasm via binding to their inhibitory subunit, IκB. After a variety of stimuli, the IκB proteins are phosphorylated, ubiquinated, and degraded, allowing for NF-κB to translocate into the nucleus where it can bind specific DNA sequences located in the promoter regions of target genes and activate gene transcription, thereby indicating its pivotal function in the regulation of immune and inflammatory responses, via the control of the transcription of inflammatory cytokine genes [10,11]. Increased activation of NF-κB has been found in macrophages and epithelial cells of patients with UC [12]. In addition, increased DNA binding activity of NF-κB that is associated with secretion of high levels of IL-1 and IL-6 is observed in macrophages from patients with UC [12,13]. These studies suggested that the activation of NF-κB plays a critical role in the initiation of intestinal inflammation of UC. From this, inhibition of NF-κB activation has been suggested as an anti-inflammatory strategy in UC.

Caspase-1 is a member of a family of caspases with large prodomains [14], and its activation is involved in apoptosis and inflammation [15]. Caspase-1 activation induces inflammation via the production of pro-inflammatory cytokines and the recruitment of neutrophils [16]. It was reported that caspase-1^−/−^ mice reduced the production of IL-6 after stimulation with lipopolysaccharide (LPS). Caspase-1 deficiency also reduced the clinical score including weight loss, diarrhea, rectal bleeding, and colon length in colitis experimental model [17]. The results of these studies demonstrated that the activation of caspase-1 is an attractive target for the treatment of inflammatory diseases. 

Chrysophanol is a member of the anthraquinone family. The results of previous pharmaceutical studies have shown that derivatives of anthraquinones exert a number of biological effects, including anticancer [18,19], hepatoprotective [20], and antimicrobial [21]. However, the exact mechanisms underlying the anti-inflammatory effects of chrysophanol remain to be thoroughly elucidated.

Dextran sulfate sodium (DSS)-induced colitis is characterized by mucosal infiltration of inflammatory cells, epithelial injury, and ulceration [22]. The pathological mechanism of DSS-induced colitis includes the production of inflammatory cytokines and mediators by macrophages that are activated after phagocytosis of DSS. The principal objective of this study was to determine whether or not chrysophanol modulates inflammatory reactions in DSS-induced colitis. Additionally, we elucidated the effect of chrysophanol in LPS-phenotype similar to human acute and chronic UC [23]. The inhibitory induced inflammatory responses in mouse peritoneal macrophages were evaluated.

## 2. Results and Discussion

### 2.1. Effect of Chrysophanol on Clinical Signs in DSS-Induced Colitis

**Figure 1:**
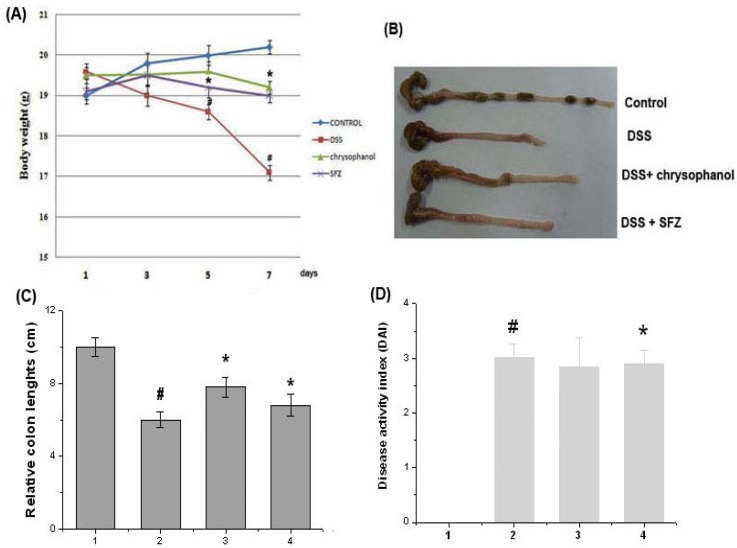
Effect of chrysophanol on clinical signs in DSS-induced colitis. Experimental colitis in mice was induced by a 5% DSS dissolved in the drinking water for 7 days. Chrysophanol was administered at doses of 5 mg/kg once a day for 7 days prior to 5% DSS supplement. (A) Body weight of mice was measured; (B) The colons were removed at day 7 after DSS treatment, and the colon lengths were measured; (C) Relative colon lengths were represented; (D) DAI was calculated as described in the Experimental. Sulfasalazine (150 mg/kg) was used as a positive control. (1) Control group; (2) DSS alone-treated group; (3) chrysophanol (5 mg/kg) + DSS treated group; (4) sulfasalazine (150 mg/kg) + DSS treated group. Values were represented in the mean ± S.E.M. (n = 5) of duplicate determinations from triplicate separate experiments(^# ^*p* < 0.05 *vs.* control, * *p* < 0.05 *vs.* DSS alone).

It was reported that in mice chrysophanol has an effect on the intestines in DSS-induced experimental colitis. As shown in Figure 1A and B, all mice treated with DSS showed the significant weight loss and colon shortening, compared to control group. However, we observed that groups administrated with chrysophanol showed the significant attenuation of body weight loss at seven days and colon shortening caused by DSS. Relative colon lengths were represented in Figure 1C. In addition, disease activity index (DAI) was determined by scoring changes in body weight, diarrhea score, and rectal bleeding score in accordance with the method described by Murthy *et al.* [24]. DAI was inhibited in groups administrated with chrysophanol compared to group of DSS (Figure 1D). 

### 2.2. Effect of Chrysophanol on Levels of Inflammatory Mediators (IL-6 and COX-2) in DSS-Induced Colitis

**Figure 2:**
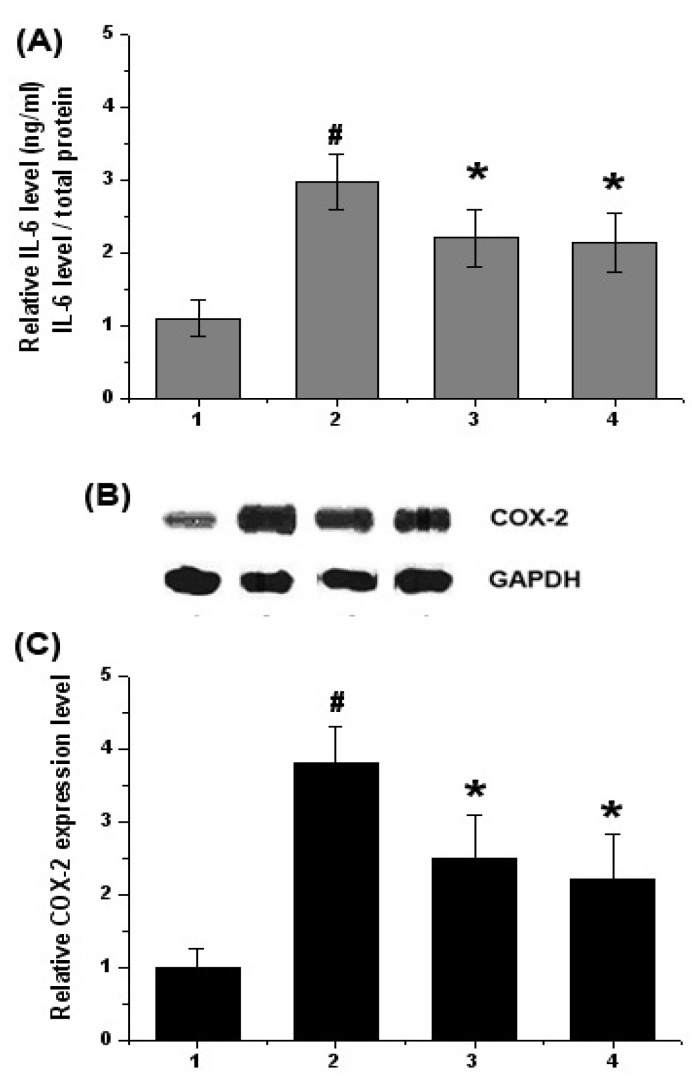
Effects of chrysophanol on the level of IL-6 and COX-2 in DSS-treated colon tissue. Experimental colitis in mice was induced by a 5% DSS dissolved in the drinking water for 7 days. Chrysophanol was administered at doses of 5 mg/kg once a day for 7 days prior to 5% DSS supplement. At the end of experiment, the colon tissues were cut out and homogenized. (A) The levels of IL-6 in the indicated groups were measured by ELISA; (B) The levels of COX-2 were evaluated by Western blot analysis; (C) The relative expression level of COX-2 was measured using an image analyzer.1) Control group; 2) DSS alone-treated group; 3) chrysophanol (5 mg/kg) + DSS treated group; 4) sulfasalazine(150 mg/kg) + DSS treated group. Values were represented in the mean ± S.E.M. (n = 5) of duplicate determinations from triplicate separate experiments (^#^*p* < 0.05 *vs.* control, *p < 0.05 *vs.* DSS alone).

We investigated the effect of chrysophanol on IL-6 production in colitis tissues. At the end of experiment, the colon tissues were homogenized, and ELISA was performed. As shown in Figure 2A, the levels of IL-6 were significantly increased in the colon tissues of DSS-treated mice compared to that of control. However, administration of chrysophanol reduced these induction induced by DSS. The inhibition rates of IL-6 production by chrysophanol were 32.11 ± 5.8%. Also, the inhibition rates of IL-6 production by SFZ were 30.01 ± 6.1%.

The effect of chrysophanol on the expression levels of COX-2 were evaluated by Western blot analysis. The expressions of COX-2 were significantly increased in the colon tissues of DSS-treated mice compared to that of control. However, administration of chrysophanol reduced the expression of COX-2 induced by DSS (Figure 2B). The relative level of COX-2 was measured using an image analyzer (Figure 2C).

### 2.3. Effect of Chrysophanol on Activation of NF-κB (p65) and Caspase-1 in DSS-Induced Colitis

**Figure 3:**
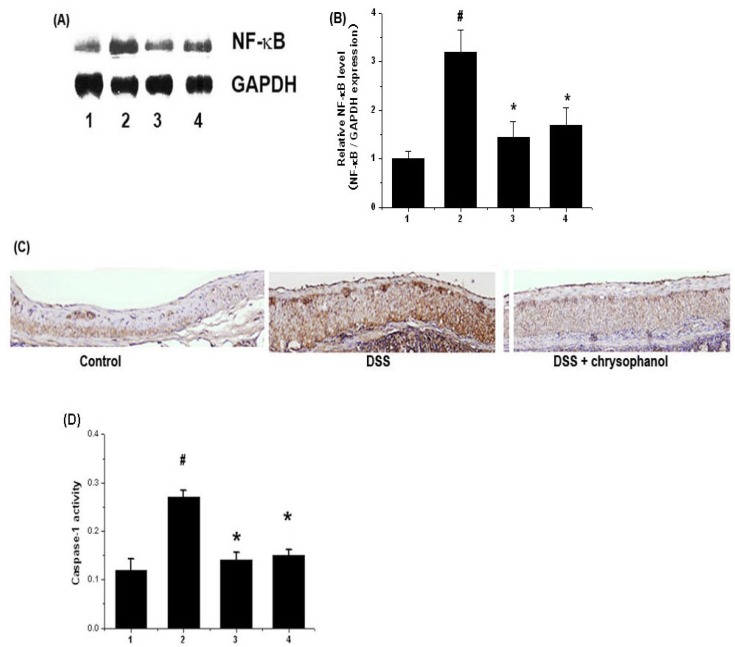
Effects of chrysophanol on the activation of NF-κB p65 and caspase-1 in DSS-treated colon tissue. At the end of experiment, the colon tissues were cut out and homogenized. (A) The levels of NF-κB p65 were evaluated by Western blot analysis; (B) The relative expression level of NF-κB was measured using an image analyzer; (C) Colon were removed and embedded in paraffin, and then 5 μm sections were prepared and stained with anti-NF-κB; (D) The enzymatic activity of caspase-1 was evaluated via a caspase colorimetric assay. 1) Control group; 2) DSS alone-treated group; 3) chrysophanol (5 mg/kg) + DSS treated group; 4) sulfasalazine (150 mg/kg) + DSS treated group.Values were represented in the mean ± S.E.M. (n = 5) of triplicate determinations from triplicate separate experiments (^# ^*p* < 0.05 *vs.* control, * *p* < 0.05 *vs.* DSS alone).

Activation of NF-κB p65 is involved in colitis, thus inhibition of NF-κB activation has been suggested as an anti-inflammatory strategy in colitis [12]. We examined whether chrysophanol regulates the activation of NF-κB p65 in colitis tissues. The activation of NF-κB p65 were significantly increased in the colon tissues (epidermis) of DSS-treated mice compared to that of control. However, administration of chrysophanol reduced significantly the activation of NF-κB (p65) induced in DSS-treated colon tissues (Figure 3A). The relative level of NF-κB was measured using an image analyzer (Figure 3B). Histology study showed that DSS dramatically induced NF-κB p65 activation, but chrysophanol diminished these increase (Figure 3C). 

The activation of caspase-1 induces inflammation via the production of inflammatory cytokines [16] and it is an attractive target for the treatment of inflammatory diseases. We also evaluated the effects of chrysophanol on caspase-1 activation using a caspase-1 assay kit. As shown in Figure 3D, the caspase-1 activity were significantly increased in the colon tissues of DSS-treated mice compared to that of control. However, administration of chrysophanol reduced these induction induced by DSS. The inhibition rate of caspase-1 activation by chrysophanol was 40.21 ± 5.8%. Also, the inhibition rates of caspase-1activation by SFZ were 37.21 ± 5.1%.

### 2.4. Effects of Chrysophanol on the Production of Cytokine, PGE_2_ and Expression of COX-2 in LPS-Stimulated Mouse Peritoneal Macrophage

**Figure 4:**
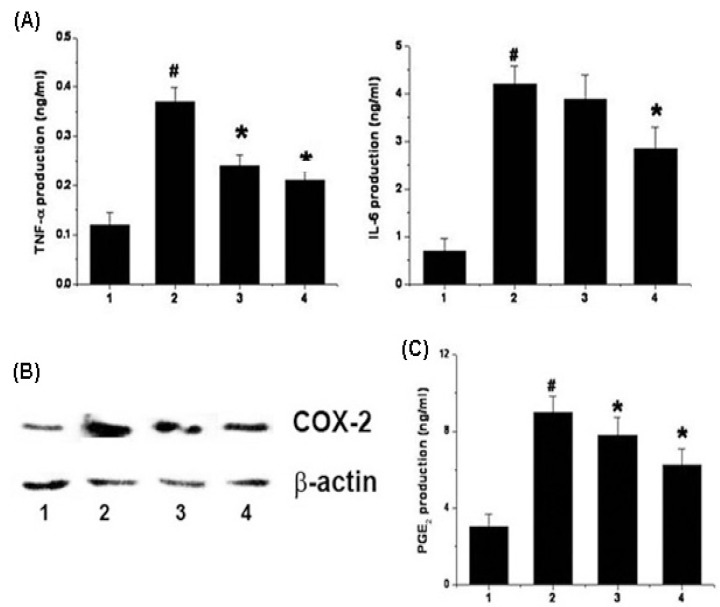
Effect of chrysophanol on TNF-α, IL-6, PGE_2_ production and COX-2 expression in LPS-stimulated murine peritoneal macrophages. (A) Cells (3 × 10^5^ cells/mL) were pretreated for 1 h with chrysophanol (2 and 20 μM), and then stimulated for 24 h with LPS (1 μg/mL). The levels of TNF-α and IL-6 in the supernatant were measured by ELISA; (B) Cells (5 × 10^6^ cells/mL) were pretreated for 1 h with chrysophanol (2 and 20 μM), and then stimulated for 24 h with LPS (1 μg/mL). The protein extracts were assayed by Western blot analysis for COX-2; (C) The amount of PGE_2_ production was measured usingimmunoassay kits. 1) unstimulated cells; 2) LPS (1 μg/mL); 3) chrysophanol (2 μM) plus LPS (1 μg/mL); 4) chrysophanol (20 μM) plus LPS (1 μg/mL). All data were represented in the mean ± S.E.M. of triplicate determinations from triplicate separate experiments (^# ^*p* < 0.05 *vs.* control, * *p* < 0.05 *vs.* LPS alone).

The effects of chrysophanol on TNF-α and IL-6 production from LPS-stimulated mouse peritoneal macrophages were evaluated. As shown in Figure 4A, the production of TNF-α and IL-6 in response to LPS was inhibited via pre-treatment with chrysophanol (2 and 20 μM). The maximal inhibition rates of TNF-α and IL-6 production by chrysophanol (20 μM) were 43.31 ± 2.8% and 37.31 ± 3.1%, respectively. No cell cytotoxicity by chrysophanol was observed (data not shown).

Western blot analysis was conducted to determine the effects of chrysophanol on LPS-induced COX-2 expression. The cells were pretreated for 1 h with chrysophanol and then treated for 24 h with LPS. As shown in Figure 4B, LPS enhanced the levels of COX-2 expression relative to that observed in un-stimulated cells. However, chrysophanol inhibited the increase in COX-2 levels. 

COX-2 catalyzes the biosynthesis of PGE_2_; therefore, we evaluated chrysophanol to determine whether or not it exerts an effect on PGE_2_ production. As shown in Figure 4C, PGE_2_ production was enhanced in response to LPS treatment; however, this increase was inhibited significantly by chrysophanol. The maximal inhibition rate of PGE_2_ production by chrysophanol (20 μM) was 40.68 ± 4.4%.

### 2.5. Effects of Chrysophanol on NF-κB and Caspase-1 Activation in LPS-Stimulated Mouse Peritoneal Macrophage

**Figure 5:**
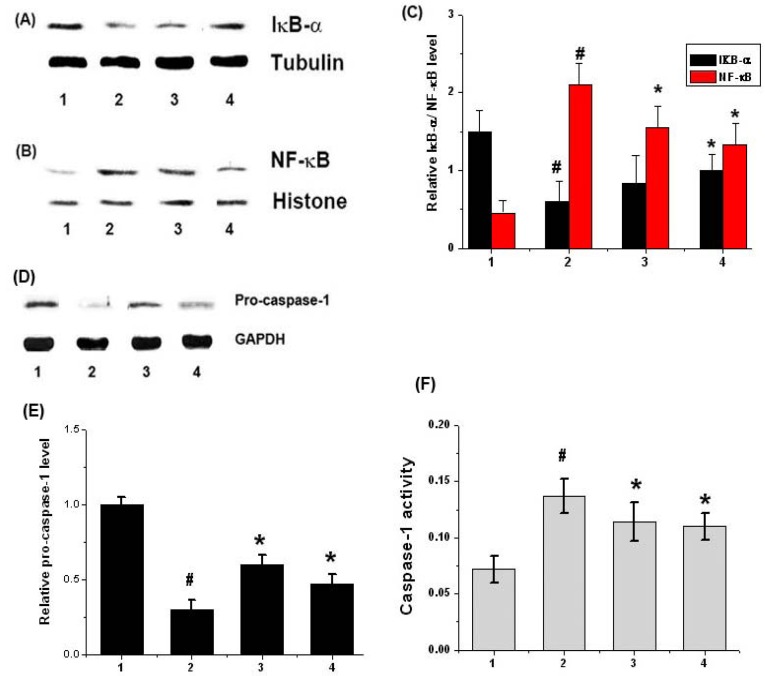
Effect of chrysophanol on degradation of IκB-α and activation of NF-κB and caspase-1 in LPS-stimulated murine peritoneal macrophages. (A) Cells (5 × 10^6^) were pretreated for 1 h with chrysophanol (2 and 20 μM) and then treated for 1 h with LPS (1 μg/mL). The cytosolic extracts were prepared as described in the Experimental section and evaluated for IκB-αby Western blot analysis; (B) Nuclear extracts were prepared as described in the Experimental section and evaluated for RelA/p65 by Western blot analysis; (C)The relative expression levels of IκB-α/ RelA/p65were measured using an image analyzer; (D) The cells were pretreated with chrysophanol (2 and 20 µM) for 1 h prior to LPS stimulation for 12 h. The levels of pro-caspase-1 were assayed by Western blot analysis; (E) The relative expression levels of pro-caspase-1 wererepresented; (F) The enzymatic activity of caspase-1 was evaluated using a caspase colorimetric assay. (1) unstimulated cells; (2) LPS (1 μg/mL); (3) chrysophanol (2 μM) plus LPS (1 μg/mL); (4) chrysophanol (20 μM) plus LPS (1 μg/mL). All data were represented in the mean ± S.E.M. of triplicate determinations from triplicate separate experiments (^# ^*P* < 0.05 *vs.* control, * *P* < 0.05 *vs.* LPS alone).

Most inhibitors of NF-κB activation exert their effects via the suppression of IκB-α degradation [25]. In order to determine whether or not the inhibitory action of chrysophanol was attributable to its effects on IκB-α degradation, the levels of IκB-α in the cytosol were assessed after LPS-stimulation via Western blot analysis. As shown in Figure 5A, we determined that LPS treatment effectively induced the degradation of IκB-α. However, chrysophanol significantly inhibited LPS-induced IκB-α degradation in mouse peritoneal macrophages. 

As NF-κB activation requires nuclear translocation of the RelA/p65 subunit of NF-κB, we assessed the effects of chrysophanol on the nuclear pool of RelA/p65 protein via Western blot analysis. In LPS-stimulated cells, the levels of Rel/p65 were increased, but chrysophanol reduced these enhanced levels of Rel/p65 in the nucleus (Figure 5B). The relative expression levels of IκB-α/ RelA/p65 were represented in Figure 5C.

Additionally, we evaluated the effects of chrysophanol on LPS-induced caspase-1 activation using Western blot analysis. The cells were pretreated for 1 h with chrysophanol and then treated for an additional 24 h with LPS. As shown in Figure 5D, LPS treatment decreased the expression of pro-caspase-1, an inactive form of caspase-1. However, this phenomenon was reduced significantly by chrysophanol treatment. The relative expression levels of pro-caspase-1 were represented in Figure 5E. We also measured the effects of chrysophanol on caspase-1 activation using a caspase-1 assay kit. We demonstrated that the enhanced caspase-1 activity was reduced significantly by chrysophanol (Figure 5F).

### 2.6. Discussion

UC is an idiopathic disease characterized by the development of intestinal inflammation [26]. Most therapies for UC include glucocorticosteroids, sulfasalazine, and so on [27,28]. However, they cause serious side effects such as hormonal disturbance, peptic ulcer, liver dysfunction, and psychological problems. In this study, we attempted to determine the effects of chrysophanol on DSS-induced colitis. Chrysophanol ameliorated the body weight loss and colon shortening caused by DSS. DAI, which was scored with three major clinical signs (weight loss, diarrhea, and rectal bleeding) was inhibited in groups administrated with chrysophanol compared to group of DSS. These results suggested that chrysophanol might inhibit effectively the symptoms of colitis caused by DSS.

At the inflammation site, the recruited cells are activated to release a host of inflammatory mediators, including TNF-α and IL-6. These mediators may contribute to the initiation and progression of the distributive inflammatory process. Therefore, the development of new biological therapies for inflammatory disease has generally focused on the blockage of members of the inflammatory cascade, such as cytokines. The results of this study revealed that chrysophanol inhibited TNF-α and IL-6 secretion in LPS-simulated mouse peritoneal macrophages. Additionally, the results showed that the levels of IL-6 and COX-2 were increased in DSS treated-colon tissues compared to normal group, but these inductions were reduced by treatment of chrysophanol in the colon tissues. These results indicate that chrysophanol exerts an anti-inflammatory effect via the regulation of pro-inflammatory cytokine levels.

NF-κB is a transcription factor that is important for the activation of many inflammatory mediators, cytokines (e. g., TNF-α and IL-6), COX-2, and the iNOS enzyme. It was reported that increased activation of NF-κB has been found in macrophages and epithelial cells of patients with UC [12]. In addition, increased DNA binding activity of NF-κB that is associated with secretion of high levels of IL-1 and IL-6 is observed in macrophages from patients with UC. Therefore, NF-κB has been recognized as an ideal target for molecular therapies employed to treat inflammatory diseases. In this study, we showed that chrysophanol inhibited the activation of NF-κB via the suppression of IκB-α degradation and Rel/p65 translocation in LPS-stimulated murine peritoneal macrophages. Additionally, we observed that the activation of NF-κB p65 were significantly increased in the colon tissues of DSS-treated mice compared to that of control. However, chrysophanol reduced this increase with DSS-treated colon tissues. Therefore, we hypothesized that chrysophanol might exert anti-inflammatory effects via the regulation of NF-κB activation. 

**Figure 6:**
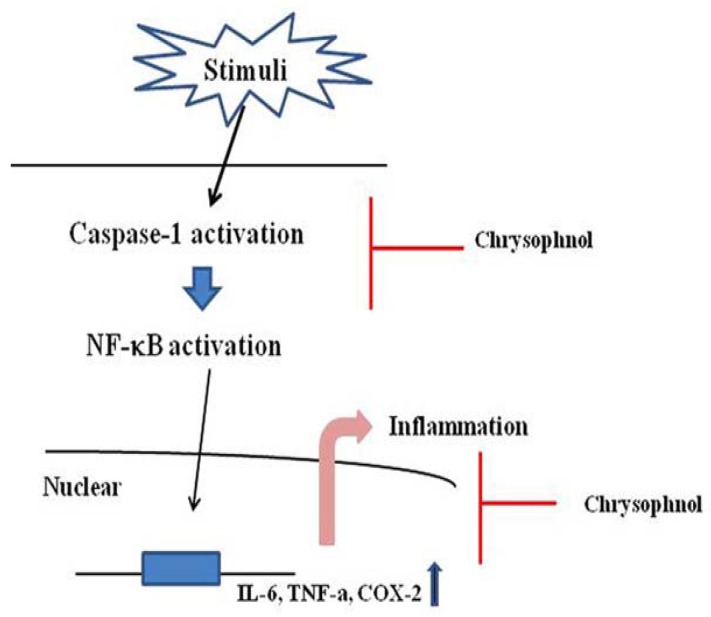
Proposed anti-inflammatory mechanism of chrysophanol in LPS-stimulated mouse peritoneal macrophage.

Caspase-1 is involved in apoptosis and inflammation. Caspase-1 activation regulates inflammation via the production of pro-inflammatory cytokines and the recruitment of neutrophils. Caspase-1 inhibitor prevented the DSS-induced colitis, and this effect is probably mediated by suppression of the inflammatory cytokines [29]. These studies indicated that the activation of caspase-1 is an attractive target for therapies for the treatment of inflammatory diseases. Therefore, we postulated that the effects of chrysophanol are mediated, at least in part, via the suppression of caspase-1 activation. In this study, we noted that chrysophanol suppressed the caspse-1 activation in LPS-stimulated murine peritoneal macrophages and DSS-induced colon tissue. This result indicated that the inhibitory effects of chrysophanol on production of inflammatory mediators might derive from the regulation of caspase-1 activation. A model of the anti-inflammatory mechanism of chrysophanol based on Lamkanfi *et al.* is provided in Figure 6 [30]. 

## 3. Experimental

### 3.1. Reagents

Chrysophanol, 3-(4,5-dimethylthiazol-2-yl)-diphenyltetrazoliumbromide (MTT) and LPS were purchased from Sigma (St. Louis, MO, USA). Anti-mouse TNF-α, biotinylated anti- mouse TNF-α and recombinant mouse TNF-α were purchased from R&D Systems (Minneapolis, MN, USA). Anti-mouse IL-6, biotinylated anti- mouse IL-6 and recombinant mouse IL-6 were purchased from Pharmingen (San Diego, CA, USA). Dulbeccos Modified Eagles Medium (DMEM), Fetal bovine serum (FBS) and thioglycollate (TG) was purchased from Gibco Laboratories (Grand Island, NY, USA). DSS (mol wt; 36,000 - 50,000) was purchased from MP Biomedicals (Solon, OH, USA). The antibodies (Ab) against COX-2, NF-κB, and β-actin were from Santa Cruz Biotechnology (Santa Cruz, CA, USA). 

### 3.2. Animals

Male C57BL/6 (6 weeks old) and female BALB/c mice (6 weeks old) were purchased from the Dae-Han Experimental Animal Center (Eumsung, South Korea), and the animals were maintained in the College of Pharmacy, Wonkwang University. The rats were housed five to ten per cage in a laminar air-flow room maintained at a temperature of 22 ± 1 °C and relative humidity of 55 ± 10% throughout the study. No animal was used more than once. Animal experimental procedures were approved by the ethics committee of Wonkwang University.

### 3.3. Peritoneal Macrophage Cultures

TG-elicited macrophages were harvested 3~4 days after i.p. injection of 2.5 mL TG to the mice and isolated, as reported previously [31]. Using 8 mL of HBSS containing 10 U/mL heparin, peritoneal lavage was performed. Then, the cells were distributed in DMEM, which was supplemented with 10% heat-inactivated FBS, in 4-well tissue culture plates (2.5 × 10^5^ cells/well) incubated for 3 h at 37°C in an atmosphere of 5% CO_2_, washed three times with HBSS to remove non-adherent cells, and equilibrated with DMEM that contained 10% FBS before treatment.

### 3.4. MTT Assay

Cell viability was determined using MTT assay. Briefly, peritoneal macrophage cell suspension (500 μL, 3 × 10^5^ cells) was cultured in 4-well plates for 24 h after treatment by each concentration of chrysophanol (2 and 20 μM). MTT solution (50 μL, 5 mg/mL) was added and then cells were incubated for 4 h at 37 °C. After washing the supernatant out, the insoluble formazan product was dissolved in DMSO. Then, optical density of 96-well culture plates was measured using enzyme-linked immunosorbent assay (ELISA) reader at 540 nm. The optical density of formazan formed in untreated control cells was taken as 100% of viability.

### 3.5. Induction of Colitis by DSS

A widely used experimental model of colitis involves oral consumption of DSS dissolved in drinking water. This produces/provokes acute and chronic colitis in rodents by inducing inflammation and recruitment of immune cells and their subsequent activation directly through epithelial cell damage and/or altered macrophage function. Acute colitis in mice (female BALB/c mice) was induced by providing drinking water containing 5% (w/v) DSS for 7 days. Mice were checked daily for loss of body weight, stool consistency and the presence of gross bleeding. Mice were randomized into groups receiving chrysophanol (5 mg/kg), sulfasalazine (150 mg/kg) as a positive control, or water as a negative control. chrysophanol and sulfasalazine were orally administrated once a day for 7 days prior to DSS treatment. Mice finally were euthanized and assessed after DSS treatment for 7 days. 

### 3.6. DAI

The activity of intestinal disease was assessed through manifestations, comprising loss of weight, diarrhea accompanied with blood and mucus, and shortening of colon [32]. As described by Murthy *et al.* [24], DAI was obtained from score of three major clinical signs (weight loss, diarrhea, and rectal bleeding). Loss of body weight was calculated as the difference between the initial and final weight. Diarrhea was defined by the absence of fecal pellet formation in the colon and the presence of continuous fluid fecal material in the colon. The appearance of rectal bleeding was separated as diarrhea containing visible blood and gross rectal bleeding and scored as described for diarrhea. DAI was calculated using the following formula: DAI = (weight loss score) + (diarrhea score) + (rectal bleeding score). The clinical parameters used here are comprehensive functional measures that are analogous to the subjective clinical symptoms observed in human ulcerative colitis [22]. This method of scoring has been validated by repeated studies. 

### 3.7. Cytokines and Prostaglandins E_2_ (PGE_2_) Assay

Cytokines assay was performed by a modified ELISA, as described previously [33]. The ELISA was devised by coating 96-well plates of mouse monoclonal Ab with specificity for TNF-α, and IL-6. Before subsequent steps in the assay, coated plates were washed with PBS containing 0.05% Tween 20. All reagents used in this assay were incubated for 2 h at 37°C. Recombinant TNF-α, and IL-6 was diluted and used as a standard. Serial dilutions starting from 10 ng/ml were used to establish the standard curve. Assay plates were exposed sequentially to biotinylated mouse TNF-α, IL-6, avidin peroxidase, and ABTS substrate solution containing 30% H_2_O_2_. The plates were read at 405 nm. The PGE_2_ level was quantified by immunoassay kits according to the manufacture’s protocols (Stressgen Biotechnologies, Ann Arbor, MI, USA).

### 3.8. Preparation of Cytoplasmic and Nuclear Extract

Nuclear and cytoplasmic extracts were prepared as described previously [34]. Briefly, after cell activation for the times indicated cells were washed with ice-cold phosphate-buffered saline (PBS) and resuspended in 60 μL of buffer A (10 mM 4-2-hydroxyethyl)-1-piperazineethanesulfonic acid (Hepes)/KOH, 2 mM MgCl_2_, 0.1 mM ethylenediaminetetraacetic acid (EDTA), 10 mM KCl, 1mM dithiothreitol (DTT), and 0.5mM phenylmethylsulfonyl fluoride (PMSF), pH 7.9). The cells were allowed to swell on ice for 15 min, lysed gently with 2.5 μL of 10% Nonide P (NP)-40, and centrifuged at 2000 g for 10 min at 4 °C. The supernatant was collected and used as the cytoplasmic extracts. The nuclei pellet was resuspended in 40 μL of buffer B (50 mM HEPES/KOH, 50 mM KCl, 300 mM NaCl, 0.1 mM EDTA, 10% glycerol, 1 mM DTT, and 0.5 mM PMSF, pH 7.9), left on ice for 20 min, inverted and the nuclear debris was spun down at 15,000 g for 15 min. The supernatant (nuclear extract) was collected, frozen in liquid nitrogen and stored at –70 °C until ready for analysis.

### 3.9. Western Blot Analysis

For analysis of the levels of COX-2, pro-caspase-1 and GAPDH, stimulated cells were rinsed twice with ice-cold PBS and then lysed in ice-cold lysis buffer (PBS containing 0.1% sodium dodecyl sulfate (SDS), 1% Triton and 1% deoxycholate). Cell lysates were centrifuged at 15,000 × g for 5 min at 4 °C; the supernatant was then mixed with an equal volume of 2 × SDS sample buffer, boiled for 5 min and then separated through a 10% denaturing protein gel. After electrophoresis, the protein was transferred to nylon membranes by electrophoretic transfer. The membranes were blocked in 5% skim milk for 2 h, rinsed and incubated overnight at 4 °C with primary antibodies (1:500). After three washes in PBS/0.5% Tween 20, the membranes were incubated for 1 h with horseradish peroxidase-linked anti-rabbit immunoglobulin (secondary antibodies). After three washes in PBS/0.1% Tween 20, the protein bands were visualized by an enhanced chemiluminescence assay following the manufacturer’s instructions (Amersham Pharmacia, Piscataway, NJ, USA) and then exposed to X-ray film.

### 3.10. Assay of Caspase-1 Activity

The enzymatic activity of caspase-1 was assayed using a caspase colorimetric assay kit according to the manufacturer's protocol (R and D systems, Minneapolis, MN, USA). The lysed cells were centrifuged at 14,000 rpm for 5 min. The protein supernatant was incubated with 50 μL reaction buffer and 5 μL caspase substrate at 37 °C for 2 h. The absorbance was measured using a plate reader at a wavelength of 405 nm. Equal amounts of the total protein from each lysate were quantified using a bicinchoninic acid protein (BCA) quantification kit.

### 3.11. Statistical Analysis

The experiments shown are a summary of the data from at least-three experiments and are presented as the mean ± SEM. Statistical evaluation of the results was performed by independent *t*-test.

## 4. Conclusions

In this study, we demonstrated first that a treatment of chrysophanol could reduce significantly the clinical signs and the levels of inflammatory mediators in a colitis model caused by DSS treatment. Additionally, the anti-inflammatory activities of chrysophanol could be attributed, at least in part, to the inhibition of proinflammatory cytokine production (TNF-α and IL-6), COX-2, and iNOS protein expression. These effects of chrysophanol are caused by the inhibition of LPS-induced NF-κB activation, IκB-α degradation, and caspase-1 activation. These results provide experimental evidence showing that chrysophanol might prove useful in the treatment of inflammatory diseases.
